# Epigenetic gestational age acceleration: a prospective cohort study investigating associations with familial, sociodemographic and birth characteristics

**DOI:** 10.1186/s13148-018-0520-1

**Published:** 2018-06-27

**Authors:** Jasmine N. Khouja, Andrew J. Simpkin, Linda M. O’Keeffe, Kaitlin H. Wade, Lotte C. Houtepen, Caroline L. Relton, Matthew Suderman, Laura D. Howe

**Affiliations:** 1 MRC Integrative Epidemiology Unit at the University of Bristol, Bristol, England; 2 Population Health Sciences, Bristol Medical School, University of Bristol, Bristol, England; 3 School of Experimental Psychology at the University of Bristol, Bristol, England

**Keywords:** DNA methylation, Epigenetics, Gestational, Age acceleration, ALSPAC, ARIES

## Abstract

**Background:**

Gestational age at delivery is associated with health and social outcomes. Recently, cord blood DNA methylation data has been used to predict gestational age. The discrepancy between gestational age predicted from DNA methylation and determined by ultrasound or last menstrual period is known as gestational age acceleration. This study investigated associations of sex, socioeconomic status, parental behaviours and characteristics and birth outcomes with gestational age acceleration.

**Results:**

Using data from the Avon Longitudinal Study of Parents and Children (*n* = 863), we found that pre-pregnancy maternal overweight and obesity were associated with greater gestational age acceleration (mean difference = 1.6 days, 95% CI 0.7 to 2.6, and 2.9 days, 95% CI 1.3 to 4.4, respectively, compared with a body mass index < 25 kg/m^2^, *p* < .001). There was evidence of an association between male sex and greater gestational age acceleration. Greater gestational age acceleration was associated with higher birthweight, birth length and head circumference of the child (mean differences per week higher gestational age acceleration: birthweight 0.1 kg, 95% CI 0.1 to 0.2, *p* < .001; birth length 0.4 cm, 95% CI 0.2 to 0.7, *p* < .001; head circumference 0.2 cm, 95% CI 0.1 to − 0.4, *p* < .001). There was evidence of an association between gestational age acceleration and mode of delivery (assisted versus unassisted delivery, odds ratio = 0.9 per week higher gestational age acceleration, 95% CI 0.7, 1.3 (*p* = .05); caesarean section versus unassisted delivery, odds ratio = 0.6, 95% CI 0.4 to 0.9 per week higher gestational age acceleration (*p* = .05)). There was no evidence of association for other parental and perinatal characteristics.

**Conclusions:**

The associations of higher maternal body mass index and larger birth size with greater gestational age acceleration may imply that maternal overweight and obesity is associated with more rapid development of the fetus in utero. The implications of gestational age acceleration for postnatal health warrant further investigation.

**Electronic supplementary material:**

The online version of this article (10.1186/s13148-018-0520-1) contains supplementary material, which is available to authorized users.

## Background

Preterm birth (≤ 37 weeks gestation) is associated with numerous health consequences such as increased mortality [1], hypertension [2, 3], insulin resistance [4] and respiratory problems in later life [5, 6]. Indeed, each additional gestational day at birth is associated with improved medical and neuropsychological outcomes in childhood [7]. Gestational age (GA) at delivery is typically determined via early obstetric ultrasound or last menstrual period (LMP), with ultrasound methods considered the more reliable [8], ‘gold standard’ procedure.

Recently, DNA methylation (DNAm) has been used to predict GA at delivery [9, 10]. This method builds on work that used DNAm to predict chronological age [11] and subsequent work, showing that the differences between predicted and chronological age are associated with disease outcomes. Those with DNAm-predicted ages that exceeded their chronological ages (age acceleration, AA) have a higher risk of cancer incidence [12–14], Alzheimer’s disease [15] and mortality [15–20]. Of note, the term AA is commonly used in the literature to describe both positive and negative differences (i.e. predicted ages above or below chronological ages), which could be misleading, but we use the term to be consistent with previous literature.

In a similar way, the gestational epigenetic clocks developed by Bohlin et al. [10] and Knight et al. [9] can be compared with actual GA to determine gestational age acceleration (GAA). There is little existing research on the association of potential predictors of GAA or the potential outcomes of GAA.

The aim of this study was to apply a previously published model for predicting GA from DNAm [10] and use this model to estimate GAA in order to (i) explore potential predictors of GAA by assessing the association of a broad range of socioeconomic variables and parental characteristics with GAA and (ii) explore potential outcomes of GAA by assessing associations of GAA with delivery and postnatal factors, using data from the Accessible Resource for Integrated Epigenomic Studies (ARIES) project, a subsample of child-mother pairs from the Avon Longitudinal Study of Parents and Children (ALSPAC).

## Results

### Study sample characteristics

The characteristics of the 863 participants from the ARIES cohort included in our analysis are displayed in Tables 1 and 2, and Additional file 1: Table S1 describes the differences between these participants and the full ALSPAC cohort from which ARIES is a subsample. Additional file 1: Table S2 shows the full range of GAs of the 863 participants included in the analysis.

**Table 1:** Characteristics of the study population (count and percent of the total sample). *N* = 863

	Count (*n*)	Percentage
Female	442	51.22
Maternal smoking during pregnancy		
Never smoker	513	60.64
Former smoker	238	28.13
Current smoker	95	11.23
Paternal smoking		
Never smoker	479	70.34
Former smoker	28	4.11
Current smoker	174	25.55
Maternal alcohol consumption during pregnancy		
Heavy/binge drinker	113	13.40
Light/moderate drinker	380	45.08
Pregnancy abstainer	308	36.54
Never drinker	42	4.98
Paternal alcohol consumption during pregnancy		
Heavy/binge drinker	583	83.05
Light/moderate drinker	97	13.82
Never drinker	22	3.13
Maternal depression (EPDS)—likely to be depressed	231	27.83
Paternal depression (EPDS)—likely to be depressed	61	9.36
Maternal pre-pregnancy BMI		
Under/normal weight	651	81.07
Overweight	114	14.20
Obese	38	4.73
Paternal BMI		
Under/normal weight	357	54.42
Overweight	250	38.11
Obese	49	7.47
Parental relationship status during pregnancy		
Married	709	83.22
Cohabiting	114	13.38
Single	29	3.40
Parity		
0	702	83.87
1	102	12.19
2+	33	3.94
Maternal age (years)		
35+	115	13.33
25–34	640	74.16
15–24	108	12.51
Paternal age (years)		
35+	219	27.17
25–34	542	67.25
15–24	45	5.58

Percentages reflect the proportion of the total sample

*EPDS* Edinburgh Postnatal Depression Scale, *BMI* body mass index

**Table 2:** Socioeconomic and perinatal characteristics of the study population (count, percent of total sample, mean and SD). *N* = 863

	Count (*n*)	%/mean (SD)
Non-manual parental social class	713	85.59
Maternal education		
Degree	172	20.28
A level	253	29.83
O level	279	32.90
Less than O level	144	16.98
Paternal education		
Degree	214	25.60
A level	252	30.14
O level	181	21.65
Less than O level	189	22.61
Housing tenure		
Owned/mortgaged	737	89.77
Rented (private)	28	3.41
Rented (council/HA)	56	6.82
Financial difficulties		
No financial difficulties	352	42.16
Some financial difficulties	347	41.56
Many financial difficulties	136	16.29
Delivery method		
Unassisted	320	60.72
Assisted	126	23.91
Caesarean section	81	15.37
Delivery complications	142	16.53
Term/post-term GA at delivery	839	97.22
APGAR score at 5 min		9.52 (0.69)
Birthweight (kg)	850	3.49 (0.49)
Birth length (cm)	735	50.77 (2.12)
Head circumference (cm)	737	34.92 (1.41)

Percentages reflect the proportion of the total sample

*HA* owned by the housing association, *APGAR* scores are based on Appearance, Pulse, Grimace, Activity, and Respiration at birth

### Modelling of GA estimates

GA was estimated from ARIES cord blood DNAm using the model of Bohlin et al. [10]. Correlation between estimated and reported GA was high (correlation coefficient *r* = 0.65) though not as high as that reported in the original publication (*r* = 0.81). Some reduction, however, was expected because the Bohlin et al. [10] model was trained and tested in distinct subsets of the same Norwegian cohort. We elected not to use the model of Knight et al. [9] because we were less confident that it would produce meaningful GAA estimates given its low correlation with GA in ARIES (*r* = 0.37) [21].

### Associations of gender, socioeconomic and parental factors with GAA

Females had lower GAA than males by 0.8 days after adjusting for sex and cell type proportion (mean difference [MD] = − 0.8 days; 95% CI − 1.4, − 0.1, *p* = .024; Table 3).

**Table 3:** Associations of gender and parental socioeconomic factors with gestational age acceleration. *N* = 863

Exposure	Mean difference in gestational age acceleration (days)		
	Mean difference	95% CI	*p*
Female	− 0.8	− 1.4, − 0.1	0.024
High/non-manual parental social class	0.1	− 0.9, 1.0	0.91
Maternal education			
Degree	(Ref)		0.72
A level	0.8	− 0.1, 1.8	
O level	− 0.1	− 1.0, 0.8	
Less than O level	− 0.3	− 1.4, 0.8	
Paternal education			
Degree	(Ref)		0.51
A level	− 0.8	− 1.6, 0.1	
O level	− 0.2	− 1.2, 0.7	
Less than O level	0.3	− 0.7, 1.2	
Housing tenure			
Owned/mortgaged	(Ref)		0.42
Rented (private)	0.7	− 1.1, 2.5	
Rented (council/HA)	0.2	− 1.1, 1.5	
Financial difficulties			
None	(Ref)		0.55
Some	− 0.1	− 0.8, 0.6	
Many	− 0.4	− 1.3, 0.6	

Results are from multiply imputed data; coefficients are mean differences adjusted for sex (except for when sex is the exposure) and cell type proportion

Maternal pre-pregnancy overweight and obese status was associated with higher GAA compared with maternal pre-pregnancy body mass index (BMI) of < 25 kg/m^2^ (MD = 1.6 days, 95% CI 0.7, 2.6 for overweight; MD = 2.9 days, 95% CI 1.3, 4.4 for obese, *p* < .001, see Table 4) after adjusting for sex, cell type proportion and parental socioeconomic factors. As GAA calculated using Bohlin et al. methods [10] is correlated with birthweight [21], we further adjusted these models including birthweight as a covariate to assess whether birthweight may be driving this effect. The results were not substantially different when additionally adjusting for birthweight (Additional file 1: Table S3).

**Table 4:** Associations between parental factors and gestational age acceleration. *N* = 863

Exposure	Mean difference in gestational age acceleration (days)					
	Model 1			Model 2		
	MD	95% CI	*p*	MD	95% CI	*p*
Maternal smoking						
Never smoker	(Ref)		0.49	(Ref)		0.50
Former smoker	0.01	− 0.8, 0.7		0.03	− 0.7, 0.8	
Current smoker	0.5	− 0.5, 1.6		0.5	− 0.7, 1.6	
Paternal smoking						
Never smoker	(Ref)		0.41	(Ref)		0.40
Former smoker	− 0.9	− 2.7, 0.9		− 0.9	− 2.7, 0.9	
Current smoker	0.1	− 0.8, 0.9		0.1	− 0.8, 0.9	
Maternal alcohol consumption						
Heavy/binge drinker	(Ref)		0.26	(Ref)		0.25
Light/moderate drinker	− 0.2	− 1.2, 0.8		− 0.3	− 1.3, 0.8	
Pregnancy abstainer	− 0.4	− 1.4, 0.7		− 0.4	− 1.4, 0.6	
Never drinker	− 1.2	− 2.9, 0.5		− 1.2	− 2.9, 0.5	
Paternal alcohol consumption						
Never drinker	(Ref)		0.54	(Ref)		0.67
Light/moderate drinker	− 0.9	− 1.9, 0.1		− 0.8	− 1.9, 0.2	
Heavy/binge drinker	0.2	− 1.8, 2.2		0.4	− 1.7, 2.4	
Maternal depression depressed	0.4	− 0.4, 1.1	0.32	0.4	− 0.3, 1.2	0.28
Paternal depression depressed	−0.5	− 1.8, 0.8	0.42	− 0.6	− 1.8, 0.8	0.49
Maternal pre-pregnancy BMI						
Under/normal weight	(Ref)		< 0.001	(Ref)		< 0.001
Overweight	1.5	0.6, 2.5		1.6	0.7, 2.6	
Obese	2.7	1.2, 4.3		2.9	1.3, 4.4	
Paternal BMI						
Under/normal weight	(Ref)		0.53	(Ref)		0.58
Overweight	0.1	− 0.7, 0.9		0.1	− 0.7, 0.9	
Obese	0.4	− 0.9, 1.8		0.4	− 1.0, 1.8	
Parental relationship status						
Married	(Ref)		0.37	(Ref)		0.47
Cohabiting	0.1	− 0.8, 1.1		0.1	− 0.9, 1.1	
Single	0.8	− 1.0, 2.5		0.7	− 1.1, 2.6	
Parity						
0	(Ref)		0.88	(Ref)		0.83
1	− 0.3	− 1.3, 0.7		− 0.4	− 1.3, 0.7	
2+	0.1	− 1.6, 1.8		0.1	− 1.6, 1.8	
Maternal age						
35+	(Ref)		0.60	(Ref)		0.69
25–34	− 0.5	− 1.5, 0.5		− 0.5	− 1.4, 0.5	
15–24	− 0.1	− 1.3, 1.2		0.03	− 1.3, 1.4	
Paternal age						
35+	(Ref)		0.27	(Ref)		0.32
25–34	− 0.4	− 1.2, 0.3		− 0.4	− 1.1, 0.4	
15–24	− 0.7	− 2.2, 0.8		− 0.6	− 2.2, 0.9	
Pregnancy complications	− 0.4	− 1.2, 0.5	0.42	− 0.6	− 1.5, 0.3	0.16

Results are from multiply imputed data; coefficients are mean differences (MD) adjusted for sex and cell type proportion (model 1) and additionally for parental social class, education, housing tenure and financial difficulties (model 2). Parental depression was measured using the Edinburgh Postnatal Depression Scale. The pregnancy complication analysis additionally adjusted for all other parental behaviour covariates

*BMI* body mass index

No clear associations were found for parental education, relationship status, smoking, alcohol consumption, depression or age, nor with housing tenure, financial difficulties, parity or pregnancy complications with GAA (Tables 3 and 4).

### Associations of GAA with delivery and birth outcomes

There were strong positive associations of GAA with birthweight (MD = 0.1 kg of birthweight per week higher GAA, 95% CI 0.1, 0.2, *p* < .001), birth length (MD = 0.4 cm of birth length per week higher GAA, 95% CI 0.2, 0.7, *p* < .001) and head circumference (MD = 0.2 cm of head circumference per week higher GAA, 95% CI 0.1, 0.4, *p* < .001) (see Table 5).

**Table 5:** Associations between gestational age acceleration and perinatal factors. *N* = 863

Outcome	Mean difference in perinatal outcome per 1 week higher gestational age acceleration					
	Model 1			Model 2		
	MD	CI	*p*	MD	CI	*p*
Birthweight (kg)	0.1	0.1, 0.2	< 0.001	0.1	0.1, 0.2	< 0.001
Birth length (cm)	0.5	0.3, 0.7	< 0.001	0.4	0.2, 0.7	< 0.001
Head circumference (cm)	0.3	0.1, 0.4	< 0.001	0.2	0.1, 0.4	< 0.001
APGAR scores (0–10)	− 0.01	− 0.1, 0.1	0.79	− 0.02	− 0.1, 0.1	0.73
	OR	CI	*p*	OR	CI	*p*
Delivery method						
Unassisted	–	(Ref)		–	(Ref)	
Assisted	0.9	0.7, 1.2		0.9	0.7, 1.3	
Caesarean section	0.7	0.5, 1.0	0.05	0.6	0.4, 0.9	0.05

Results are from multiply imputed data; coefficients are mean differences (MD) or odds ratios (OR) adjusted for sex and cell type proportion in model 1 and additionally adjusted for parental social class, education, smoking, alcohol use, depression, body mass index, age and relationship status as well as housing tenure, financial difficulties and parity in model 2. APGAR scores are based on Appearance, Pulse, Grimace, Activity, and Respiration at birth

No association was observed between GAA and Appearance, Pulse, Grimace, Activity, and Respiration (APGAR) score at 5 min. GAA was inversely associated with assisted delivery (excluding caesarean section) (odds ratio [OR] = 0.9 per week higher GAA, 95% CI 0.7, 1.3, see Table 5) and caesarean section (OR = 0.6 per week higher GAA, 95% CI 0.4, 0.9, *p* = .05).

There were no substantial differences between the imputed and observed data sets (Additional file 1: Table S4). Adjusting for cell type proportion did not substantially alter our results (see Additional file 1: Tables S5 to S7 for unadjusted results) and neither did adjustment for socioeconomic status (SES) and other potential confounders.

## Discussion

Our analyses indicated that male sex, higher maternal pre-pregnancy BMI and vaginal delivery are associated with higher GAA. Our results also indicated that higher GAA was associated with birth size (birthweight, birth length and head circumference). There was no clear evidence of any associations of GAA with parental education, relationship status, smoking, alcohol consumption, depression or age, nor with housing tenure, financial difficulties, parity, pregnancy complications or APGAR scores.

Unlike in AA research, where AA has been associated with negative outcomes such as all-cause mortality and Alzheimer’s disease [15–20], it is currently unclear whether accelerated GA at birth is beneficial or detrimental to a fetus or newborn. From previous research using ARIES data, we know sex, birthweight, caesarean section and maternal BMI have also been associated with AA [22]. A recent study has also shown associations of GAA with birth size and sex [23]. However, in their main analysis, associations with birth size were in the opposite direction to our analysis when the raw GAA-GA difference was used as the outcome, i.e. higher GAA was associated with smaller size at birth. When calculating GAA using the residuals from a regression of DNAm-predicted GA on reported GA, as GAA was calculated in this study, there was no clear association between GAA and size at birth. In further contrast, we did not replicate their associations with maternal age, APGAR scores (at 1 min) and pregnancy complications (pre-eclampsia). The discrepancies in the results could be related to the focus on raw differences rather than residuals in the main analysis of Girchenko et al. [23]. We did not estimate GAA as the difference between DNAm-predicted GA and reported GA because the confounding effect of GA is not accounted for in this approach, whereas the residual-based approach ensures GAA is uncorrelated with GA.

Another explanation for the discrepancies between our findings and the findings of Girchenko et al. [23] is their use of the Knight et al. [9] GA prediction model rather than the Bohlin et al. [10] model applied in this study. We have previously noted several key methodological differences in the derivation of the Knight et al. [9] and the Bohlin et al. [10] models that influence the accuracy of the GA prediction in this cohort [21], such as the inclusion of preterm infants in the test set of the Knight et al. model [9] which is inappropriate for a data set with few pre-term births (as in this study). Additionally, the number of CpGs (148) included in the Knight et al. [9] training model was close to the sample size of the training set (207) and the model was then tested with a much larger sample size which may have resulted in an overfitting of the model. In contrast, the number of CpGs (96) in the Bohlin et al. [10] training model was much lower than the sample size of the training set (1068). Thus, the Bohlin et al. [10] model provided us with the best estimate of GAA for this cohort [21], which was reflected in the stronger correlation found between reported GA in the ARIES cohort and the model predictions of GA compared to the Knight et al. [9] model. The Bohlin et al. [10] model for prediction of GA from DNAm performed well in our data, adding support to the notion that DNAm could potentially be used as a marker of GA in data sets where GA has not been measured.

In this study, we were able to apply the epigenetic clock created by Bohlin et al. [10] in ARIES data. Despite the original model being trained only on a Norwegian cohort, the methods of Bohlin et al. [10] transferred to a UK cohort with considerable accuracy (*r* = 0.65). Additionally, the use of ARIES and ALSPAC data (a large and rich source of longitudinal data for children and their families) allowed us to assess the associations of GAA with a wide range of socioeconomic, parental and perinatal factors. The longitudinal nature of ARIES also enabled comparisons between GAA and AA at multiple ages.

Although the ARIES sub-sample is a more affluent sample of the full ALSPAC cohort, our results were robust to adjustment for SES. This is in line with some evidence that such differences are unlikely to severely bias association studies [24–27]. To account for missing data, we used multiple imputation, which maximised our statistical power. The results were consistent when using complete-case observed data.

Following Gervin et al.’s [28] methods, the regression analyses were adjusted for cell type proportions, even though GA is associated with variation in cell type proportions and this adjustment could therefore potentially bias the results. However, comparing the data before and after adjustment, there were no substantial differences in the results. Additionally, only weak associations were found between GA and cell type composition in this study (Additional file 1: Figure S1). Another potential issue with adjusting for cell type composition arises from deriving cord blood references from full-term births only; cord blood cell counts may be inaccurate for cord blood methylation profiles of preterm infants. However, a small proportion of the participants in our study were pre-term (3%) so it is unlikely that this will have substantially impacted the results.

Interestingly, there is little overlap between probes used in the Bohlin et al. [10] model of GA predication and the Horvath [11] model of age prediction, with only one CpG site (cg08965235 in the latent-transforming growth factor beta-binding protein 3 gene) overlapping between the models. In contrast to the Horvath [11] model, which compares accurate measures of chronological age and epigenetic predictions, the Bohlin et al. [10] model compares potentially inaccurate LMP/ultrasound estimates to epigenetic predictions. Consequently, inaccurate GA estimates may have impacted upon the GAA estimates in this research, especially as the majority of the GA estimates in ALSPAC are derived from LMP, since ultrasound estimation of GA was not common at the time of recruitment. Additionally, the GA predictions using the Bohlin et al. [10] model were more accurate using ultrasound methods rather than LMP. This may mean that our estimates may not be as accurate as if ultrasound GAs had been used and this could explain the discrepancy in accuracy between the Bohlin et al. [10] test data and the ARIES data, as detailed by Simpkin and colleagues [21].

## Conclusions

Our results suggest that higher maternal BMI is strongly associated with higher GAA and that higher GAA is strongly associated with larger size at birth (birthweight, birth length and head circumference). In addition, we found weaker associations of sex and delivery method with GAA. Our results may indicate that having a BMI over 25 kg/m^2^ is associated with more rapid development of the fetus in utero. The implications of GAA for postnatal growth, development and health warrant further investigation.

## Methods

### Study population

This study used DNAm data generated under the auspices of the ALSPAC [29, 30]. ALSPAC recruited 14,541 pregnant women with expected delivery dates between April 1991 and December 1992. Of these initial pregnancies, there were 14,062 live births and 13,988 children who were alive at 1 year of age. The study website contains details of all the data that are available through a fully searchable data dictionary (http://www.bris.ac.uk/alspac/researchers/data-access/data-dictionary).

As part of the ARIES [31] project (http://www.ariesepigenomics.org.uk), a sub-sample of 1018 ALSPAC child-mother pairs had DNAm measured using the Infinium HumanMethylation450 BeadChip (Illumina, Inc.) [32]. The ARIES sub-sample was selected based on availability of DNA samples at three time points (birth, mean 7.5 years and mean 15.5 years). DNAm was measured three times in ALSPAC offspring, from cord blood at birth and from peripheral blood at approximately ages 7 and 17.

### Laboratory methods, quality control and pre-processing

All DNAm wet-lab and pre-processing analyses were performed at the University of Bristol as part of the ARIES project. Following extraction, DNA was bisulphite converted using the Zymo EZ DNA MethylationTM kit (Zymo, Irvine, CA). Infinium HumanMethylation450 BeadChips were used to measure genome-wide DNAm levels at over 485,000 CpG sites. The arrays were scanned using an Illumina iScan, with initial quality review using GenomeStudio. The level of DNAm is expressed as a ‘beta’ value (*β* value), ranging from 0 (no cytosine methylation) to 1 (complete cytosine methylation). *β* values are reported as percentages. Several quality control steps were included in the laboratory pipeline which are described in detail elsewhere [33].

### Epigenetic GA prediction

Using a recently published model [10], we derived epigenetic gestational age (EGA) from cord blood DNAm. The Bohlin et al. [10] model was chosen over the Knight et al. [9] model due to its much stronger correlation with GA in ARIES (*r* = 0.65 compared to *r* = 0.37). This epigenetic clock for GA at delivery uses 96 CpG sites to predict GA from cord blood methylation. We obtained GAA as the residuals from a regression of EGA on observed GA. GA was gathered from clinical records and determined by LMP for the majority; however, on some occasions, this measure was updated following a dating ultrasound. It is not known for which individual GA was based on LMP or ultrasound but as updating GA based on ultrasound was not common practice at the time of the measurement, the numbers are likely to be low. To be consistent with previous literature, we have used the terms ‘AA’ and ‘GAA’ to describe both positive and negative differences (i.e. predicted ages above or below chronological/gestational ages). A positive GAA corresponds to an EGA that was higher than actual GA and vice versa.

### Socioeconomic, parental and perinatal characteristics

Socioeconomic factors included housing tenure, social class, parental education and financial difficulties. Parental factors included parental smoking, alcohol use, mental health, relationship status, BMI and age. Finally, perinatal variables considered were child’s sex, birthweight, birth length, head circumference and APGAR score at 5 min as well as the occurrence of any pregnancy complications and the delivery type. All variables were measured through questionnaires at different times during pregnancy (socioeconomic and parental variables), by trained ALSPAC staff shortly after birth (anthropometry at birth) or from obstetric records (pregnancy complications, child’s sex and APGAR score). Full details of measurement of these factors are in Additional file 1.

### Statistical analysis

Sex, SES, parental behaviours and pregnancy complications were analysed as potential determinants of GAA. Associations between these factors and GAA were assessed in linear regression models with GAA as the outcome. Models with parental behaviours as the exposure were adjusted for SES variables as confounders. Birth size, delivery method and APGAR score were considered as potential outcomes of GAA. Associations of GAA with these factors were assessed using linear or multinomial logistic regression as appropriate, with GAA as an exposure and parental behaviour and SES variables included as potential confounders. We performed the analysis in this way due to the temporal ordering of the variables, although we do not necessarily hypothesise a direct causal effect of GAA on these outcome variables. The associations were analysed in two models: (1) adjusted for sex and cell type proportion and (2) with additional adjustment for potential confounders, as appropriate for the specific model. The Gervin et al. [28] methods were used for cell-type proportion estimations. Due to missingness in the observed data set, analyses were completed using 100 multiply imputed data sets (see Additional file 1: Table S8 for further information). There were no substantial differences between the analysis of the observed data and the multiply imputed data sets.

## Additional file

Additional file 1: **Tables S1–S8.** Supplementary methods. Details of measurement of socioeconomic, family and perinatal variables and supplementary tables. (DOCX 671 kb)

## Abbreviations

AA: Age acceleration
ALSPAC: Avon Longitudinal Study of Parents and Children
APGAR: Appearance, Pulse, Grimace, Activity, and Respiration
ARIES: Accessible Resource for Integrated Epigenomic Studies
BMI: Body mass index
DNAm: DNA methylation
EGA: Epigenetic gestational age
EPDS: Edinburgh Postnatal Depression Scale
GA: Gestational age
GAA: Gestational age acceleration
HA: Owned by the housing association
LMP: Last menstrual period
MD: Mean differences
OR: Odds ratio
SD: Standard deviation
SES: Socio-economic status
