# Supplementary material for: Epigenetic gestational age acceleration: a prospective cohort study investigating associations with familial, sociodemographic and birth characteristics
Source: Clin Epigenetics. 2018 Jun 27;10:86. doi: 10.1186/s13148-018-0520-1 (PMC6020346; doi:10.1186/s13148-018-0520-1)
Supplement: Supplementary file 1 — Tables S1–S8. Supplementary methods. Details of measurement of socioeconomic, family and perinatal variables and supplementary tables. (DOCX 671 kb) [file 13148_2018_520_MOESM1_ESM.docx]

We thank the reviewers and the editors for this good news. We can confirm that the text is correct; gestational age was included in the imputation models as a binary variable (pre term versus term). Gestational age was only included in Table 1 (as a binary measure) to describe the cohort, and was not used in our main analyses. We do not think this will have made a difference to any of our analyses, especially as: i) there was no missing data for gestational age for participants included in our analysis, and ii) gestational age acceleration is derived from a regression of DNAm gestational age on gestational age, and is therefore by definition independent of gestational age.
